# GINS1 Induced Sorafenib Resistance by Promoting Cancer Stem Properties in Human Hepatocellular Cancer Cells

**DOI:** 10.3389/fcell.2021.711894

**Published:** 2021-08-03

**Authors:** Sheng Li, Lina Wu, Hong Zhang, Xijuan Liu, Zilei Wang, Bin Dong, Guang Cao

**Affiliations:** ^1^Key Laboratory of Carcinogenesis and Translational Research (Ministry of Education/Beijing), Department I of Thoracic Oncology, Peking University Cancer Hospital and Institute, Beijing, China; ^2^Key Laboratory of Carcinogenesis and Translational Research (Ministry of Education/Beijing), Central Laboratory, Peking University Cancer Hospital and Institute, Beijing, China; ^3^Key Laboratory of Carcinogenesis and Translational Research (Ministry of Education/Beijing), Department of Interventional Therapy, Peking University Cancer Hospital and Institute, Beijing, China

**Keywords:** hepatocellular carcinoma, cancer stem cell, GINS1, sorafenib, drug resistance

## Abstract

Hepatocellular carcinoma (HCC) is characterized by a high rate of incidence and recurrence, and resistance to chemotherapy may aggravate the poor prognosis of HCC patients. Sorafenib resistance is a conundrum to the treatment of advanced/recurrent HCC. Therefore, studies on the molecular pathogenesis of HCC and the resistance to sorafenib are of great interest. Here, we report that GINS1 was highly expressed in HCC tumors, associated with tumor grades, and predicted poor patient survival using Gene Expression Omnibus (GEO) databases exploration. Cell cycle, cell proliferation assay and *in vivo* xenograft mouse model indicated that knocking down GINS1 induced in G1/S phase cell cycle arrest and decreased tumor cells proliferation *in vitro* and *in vivo*. Spheroid formation assay results showed that GINS1 promoted the stem cell activity of HCC tumor cells. Furthermore, GEO database (GSE17112) analysis showed that HRAS oncogenic gene set was enriched in GINS1 high-expressed cancer cells, and quantitative real-time PCR, and Western blot results proved that GINS1 enhanced HCC progression through regulating HRAS signaling pathway. Moreover, knocking down endogenous GINS1 with shGINS1 increased the sensitivity of HCC cells to sorafenib, and restoring HRAS or stem associated pathway partly recovered the sorafenib resistance. Overall, the collective findings highlight GINS1 functions in hepatocarcinogenesis and sorafenib resistance, and indicate its potential use of GINS1 in drug-resistant HCC.

## Introduction

Hepatocellular carcinoma (HCC) is one of the most frequently diagnosed cancer and the fifth leading causes of cancer-related deaths worldwide (Llovet et al., 2016). Due to the late diagnosis and the resistance to conventional chemotherapeutic drug, HCC patients at advanced stage usually survived no more than 1 year (Llovet et al., 2016). With the development of cancer molecular therapy, a multi-kinase inhibitor Sorafenib becomes the standard therapy for patients with advanced-stage HCC (Ikeda et al., 2018; Marisi et al., 2018; He et al., 2019). Although advance in diagnosis followed by systemic examination and the developmental system treatment, the prognosis of advanced-HCC patients is dismal (Boland and Wu, 2018). The molecular pathogenesis of HCC and the resistance to chemotherapeutic drugs is mandatory to be resolved.

Dysregulation of cell cycle-related proteins, leading to unrestrained proliferation, has been shown to be a hallmark of HCC development and progression (Pascale et al., 2002). GINS1, is a member of GINS (Go-Ichi-Nii-San) complex, which plays a vital role in the initiation and elongation of DNA replication (Bauerschmidt et al., 2007). GINS1 is mainly involved in early embryogenesis in physiological condition (Ueno et al., 2005). Moreover, GINS1 participated in tumorigenesis in pathological condition. High expression of GINS1 was found in breast and lung cancer, and was related to enhanced ability in tumor proliferation and poor patient prognosis (Nakahara et al., 2010; Zhang et al., 2015).

In current study, GINS1 was identified to be highly expressed in HCC through Gene Expression Omnibus (GEO) databases exploration. High GINS1 expression predicted poor patient survival. Then GINS1 functions in hepatocarcinogenesis and sorafenib resistance were further investigated.

## Materials and Methods

### Microarray and Bioinformatics Analysis

The gene expression profiles of GSE14520 submitted by Wang et al. were downloaded from GEO database. GSE14520 were based on two platforms. Tumor and paired non-tumor samples of 22 patients of cohort 1 were carried out on Affymetrix GeneChip HG-U133A 2.0 arrays (Affymetrix, Santa Clara, CA, United States) according to the manufacturer’s protocol. 42 paired tumor and non-tumor samples of cohort 2, and 183 tumors and 179 non-tumors samples of cohort 2 were processed on the 96 HT HG-U133A microarray platform. The analysis of the raw data files (.cel) was performed using Partek Genomics Suite (version 3.0, Broad Institute, United States). We applied PCA analysis to identify each cohort of the tumor and normal groups. MAS5 were employed to do DEGs. Then, we used a Benjamini-Hochberg Procedure to identify DEGs with a defined *P* value with FDR cutoff of <0.05 as statistically significant. Hierarchical clustering analysis was used to categorize the data into two groups that had similar expression patterns between tumors and normal samples. To analyze the DEGs at the functional level, gene ontology enrichment was performed using Partek Genomics Suite. GSE17112 and GSE143233 were downloaded from GEO database and subsequently analyzed by RVersion 3.5,^1^ with edgeR package. Fold change (FC) of gene expression was calculated with a cut-off of log2FC < –1, log2FC ≥ 1, and *P* value < 0.05. Gene Set Enrichment Analysis (GSEA) was used from website tools^2^ (Subramanian et al., 2005). Cancer cell lines sample information, including gene expression, drug sensitivity were retrieved from the Cancer Cell Line Encyclopedia (CCLE) project and downloaded from Broad Institute DepMap web portal (https://depmap.org/portal/download/). *P* < 0.05 was considered statistically significant.

### Cell Cultures and Reagents

Human liver cancer cell lines, HepG2, PLC, and HepB3 were obtained from American Type Culture Collection (Manassas, VA, United States). Huh7 was obtained from the Japan Society for the Promotion of Science (Tokyo, Japan). These cells were maintained in 1,640 medium (Gibco, Thermo Fisher, Waltham, MA, United States) supplemented with 10% heat-inactivated fetal bovine serum (Hyclone) and penicillin/streptomycin at 37°C in a humidified atmosphere of 5% CO2.

The reagents used in this study were: Antibodies of anti-GINS1 (ab181112), anti-BMI1 (ab126783), and anti-RAS (ab52939) were purchased from Abcam (Cambridge, United Kingdom). KLF4 (D1F2) and glyceraldehyde 3-phosphate dehydrogenase (GAPDH; D16H11) were obtained from Cell Signaling Technology (Danvers, MA, United States).

### Construction of GINS1 shRNA-Containing Lentivirus and Infection

The sequences of shGINS1-1, shGINS1-2, and non-silencing control shRNA (shCon) were 5′-GATCCGCTTGCCAAATGCATTACGATTTCAAGAGAAATC GTAATGCATTTGGCAAGTTTTTT-3′, 5′-GATCCGGAGGAG ATGAAAGCTTTGTATTTCAAGAGAATACAAAGCTTTCAT CTCCTCTTTTTT-3′, and 5′-GATCCGCGAGAAGCGCGAT CACATGTTCAAGAGACATGTGATCGCGCTTCTCGTTTTT T-3′, respectively. The nucleotide sequences were inserted into the plasmid through the pFH-L vector (Shanghai Hollybio, China) and the generated lentiviral-based shRNA-expressing vectors were confirmed by DNA sequencing. Lentiviruses were generated by transfection of 293 T cells at 80% confluence with generated plasmids. The cells were starved for 2 h before transfection, and shGINS1-1, shGINS1-2, or -shCon and the packaging vector carriers pVSVG-I and PCMVΔR8.92 were transfected into cells using Lipofectamine 2000. The supernatant was collected 48 h after transfection and lentiviral particles were harvested by ultracentrifugation (4,000 g) at 4°C for 10 min. The collected virus particles were filtered through a 45 μM filter and the filtrate was centrifuged (4,000 g at 4°C) for 15 min to collect the viral concentrate.

Huh7 or HepG2 cancer cells were then infected with GINS1 shRNA- or control shRNA-expressing lentiviruses. The cells were seeded (5 × 10^4^ cells per well) in six-well plates, and after 24 h of incubation, the culture medium was replaced with Opti-MEM medium containing the appropriate amount of the virus. The cells were then incubated with the virus for another 48 h.

### RNA Isolation and Quantitative Real-Time PCR

Total RNA was isolated from cells using the Trizol reagent (Invitrogen, Carlsbad, CA, United States). First-strand cDNA was synthesized from total RNA using oligo dT (Nagahama et al., 2010) primers using moloney murine leukemia virus reverse transcriptase (MMLV; Invitrogen). Quantitative real-time PCR was performed using SYBR Green PCR Master Mix (Toyobo, Osaka, Japan) on ABI 7500 System (Applied Biosystems, Foster City, CA, United States). The expression of the target gene was normalized to that of GAPDH. FC was calculated by the 2^–ΔΔCt^ method where ΔCt = Ct(Target) – Ct(Reference). The sequences of primers were listed in Table 1.

**TABLE 1:** Primers used for RT-qPCR.

Primer name	Forward primers	Reverse primers
BMI1	5′-ATGAGGAGGAACCTTTAAA GGA-3′	5′-GAAGTGGACCATTCCTTCTC-3′
GAPDH	5′-AATCCCATCACCATCTTCC-3′	5′-CATCACGCCACAGTTTCC-3′
GINS1	5′-AAAGTCAGGTGGACGAAG-3′	5′-CATTTGGCAAGACGCTAC-3′
HRAS	5′-CGGAATATAAGCTGGTGGTG-3′	5′-GGTAGGAATCCTCTATAG TGGG-3′
KLF4	5′-CCCACACTTGTGATTACGC-3′	5′-GGTAAGGTTTCTCACCTGTG-3′
LIF	5′-AGTGCCAATGCCCTCTTTAT-3′	5′-ACACGACTATGCGGTACAGC-3′
SKP2	5′-TATGCACAGGAAGCACCTC-3′	5′-TCTTGCTGGAATCCCATCC-3′
SOX2	5′-CGGATTATAAATACCGGCCC-3′	5′-GTGTACTTATCCTTCTTCATG AGC-3′

### Western Blot Analysis

For immunoblot analysis, cells were harvested and lysed in RIPA lysis buffer (WB0002, TDYBio, Beijing, China). Protein concentrations were determined using the BCA protein assay kit (Thermo Fisher, Waltham, MA, United States). Protein samples (20 μg per lane) were separated on the 8–15% SDS-polyacrylamide gel electrophoresis and transferred onto polyvinylidene difluoride membranes (Immobilon-P, Millipore, Bedford, MA, United States). Then the membranes were blocked in 5% non-fat milk or bovine serum albumin (for phosphorylated proteins) in phosphate-buffered saline (PBS) with 0.1% Tween-20, probed with primary antibodies overnight at 4°C. After washing, the membranes were incubated with appropriate horseradish peroxidase-conjugated secondary antibodies. Visualization of the protein bands was accomplished using an Immobilon Western Chemiluminescent HRP substrate (Millipore). Image J software was used to calculate the expression of each protein, which was normalized by GAPDH.

### Cell Proliferation Assays and Cell Viability Assay

Cells were seeded into 96-well plates (2,000 cells/well, 5 wells/group) and cultured for at least 96 h to determine the proliferation curves. The cells were photographed every 6 h in the long-term dynamic observation platform (IncuCyte, Essen, MI, United States). The cell confluence was analyzed using IncuCyte ZOOM software (Essen, Ann Arbor, MI, United States). The cytotoxicity of sorafenib was determined by cytotoxic MTT assay. Cells were suspended in complete RPMI-1640 medium and plated at a density of 5 × 10^3^ cells/well in 96-well culture dishes (Costar, Cambridge, MA, United States). Different concentrations of sorafenib were added into each plate with 0.5% (v/v) final concentration of DMSO in all wells and incubated for an additional 72 h. For collection time points, cells were incubated with MTT at 37°C for 4 h, and the precipitate was dissolved in DMSO. Subsequently, the absorbance (optical density, OD) at 570 nm was measured using a microplate reader (Model 680; Bio-Rad Laboratories, Hercules, CA, United States). At least three independent experiments were performed to obtain the IC50 values, calculated using the fitted concentration–response curve of each drug regimen.

### Cell Cycle Assay

Cells were trypsinized, collected and washed twice with cold PBS. Cells were fixed in cold 75% ethanol for overnight at 4°C. Fixed cells were pelleted and stained in the propidium iodide (PI) solution (50 μg/ml PI, 50 μg/ml RNase A, 0.1% Triton X-100, and 0.1 mM EDTA) in the dark at 4°C for 15 min. Flow cytometric quantification of DNA were performed by a CytoFLEX S, and operated using CytExpert Software (Beckman Coulter, Brea, CA, United States).

### Spheroid Formation Assay

For tumor sphere formation, 100 cells/well were plated in serum-free DMEM/F12 medium (Invitrogen, Thermo Fisher, Waltham, MA, United States), supplemented with 2% B27 (Invitrogen), 20 ng/ml bFGF (Peprotech, Rocky Hill, NJ, United States), 10 ng/ml HGF (Peprotech), and 1% methylcellulose (Sigma) into Ultra-low attachment 96-well plate (Corning, NY, United States) for 14 days. Individual spheres ≥ 50 μm in each replicate well were counted under a microscope (Olympus, Shinjuku, Japan).

### *In vivo* Xenograft Mouse Model

Four to six weeks female BALB/c nude mice (Vital River Laboratories, Beijing, China) were housed and maintained in the SPF-level animal laboratory of Peking University Cancer Hospital and Institute. All animal experiments were carried out in accordance with the National Institutes of Health Guide (Guide for the Care and Use of Laboratory Animals, 2011) and approved by the Animal Experimental Ethics Committee of Peking University Cancer Hospital and Institute. 100 μL of Huh7 cell w/o GINS1 ablation were injected subcutaneously (1 × 10^7^/mL in each mouse, random 5 mice for each group). The tumor size were measured twice per week for 4 weeks, and tumor volume was calculated according to the equation: length × (width)^2^/2. Tumor xenografts were separated and measured at the end point.

### Survival Analysis

The Kaplan–Meier plotter^3^ is an online tool applied to assess the effect of 54k genes on survival in 21 cancer types. The hepatocellular cancer mRNA database in the Kaplan–Meier plotter was applied to evaluate the prognostic values of GINS1, CKS2, and TOP2A genes in HCC patients. For each gene, cancer patients were divided into two groups according to the median values of mRNA expression. *P* < 0.01 was considered to indicate a statistically significant result.

### Statistical Analysis

For continuous variables, the data were expressed as the means ± standard deviation (SD). All data were first tested using Shapiro–Wilk test (test of normality) and *F*-test (test for homogeneity of variance), followed by Student’s *t*-test or Mann Whitney *U* test to determine the difference between two independent groups. Median expression values of genes were used as cutoff value to discriminate high and low expression. Overall survival curves were plotted according to the Kaplan–Meier method and the generalized log-rank test was applied to analyze the survival curves. Experiments were done in triplicate. All data were analyzed with SPSS statistical software 16.0 (SPSS Inc., Chicago, IL, United States) and Graphpad prism 8 software (GraphPad Software, La Jolla, CA, United States). *P* < 0.05 was considered statistically significant difference between values.

## Results

### GINS1 Significantly Correlates With the Poor Survival of HCC

**FIGURE 1:**
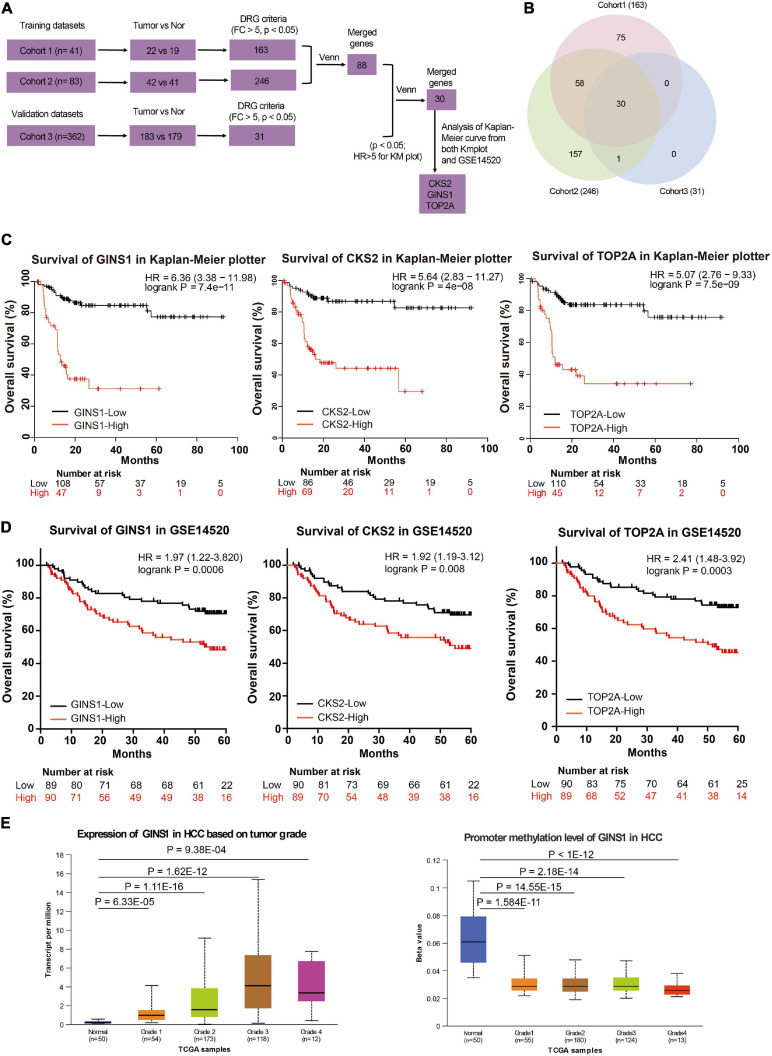
Transcriptome and survival analysis in the two independent databases. **(A,B)** gene expression profiles and venn diagram in GSE14520. **(C,D)** Survival analysis present within Kaplan–Meier plotter online tool **(C)** and the GSE14520 data **(D)**. **(E)** transcriptional (left panel) and promoter methylation (right panel) levels of GINS1 in HCC based on tumor grade present within TCGA data. Data are presented as mean ± SD.

GSE14520 database in GEO was used to evaluate the correlation between gene expression and overall survival. This query was conducted on 41 samples (cohort 1) and 83 samples (cohort 2) as training groups and 362 samples as testing group available in U133A platform with the GSE14520 database. 163 genes (from cohort 1) and 246 genes (from cohort 2) were up-regulated (fold change, FC > 5, *P* < 0.05) in the training group. Then 31 significantly up-regulated genes were identified (Figure 1A, FC > 5, *P* < 0.05) in the testing group. Collectively from the three groups, 30 genes showed consistent up-regulation (Figure 1B and Supplementary Table 1). The expression of 3 genes out of these 30 genes, including CKS2, GINS1, and TOP2A showed a significant inverse correlation with HCC overall survival in both Kaplan–Meier plotter online tool and GSE14520 (Figures 1C,D, *P* < 0.05). Furthermore, GINS1 showed the most significant up-regulation in the late-phase of HCC accompanied with a promoter hypo-methylation when compared with the adjacent normal liver tissues (Figure 1E; Chandrashekar et al., 2017). The results indicated that GINS1 may function as a key player in HCC progression. Therefore, GINS1 was selected for further investigation.

### GINS1 Induces the Cell Cycle and Promotes the Proliferation of HCC Cells

**FIGURE 2:**
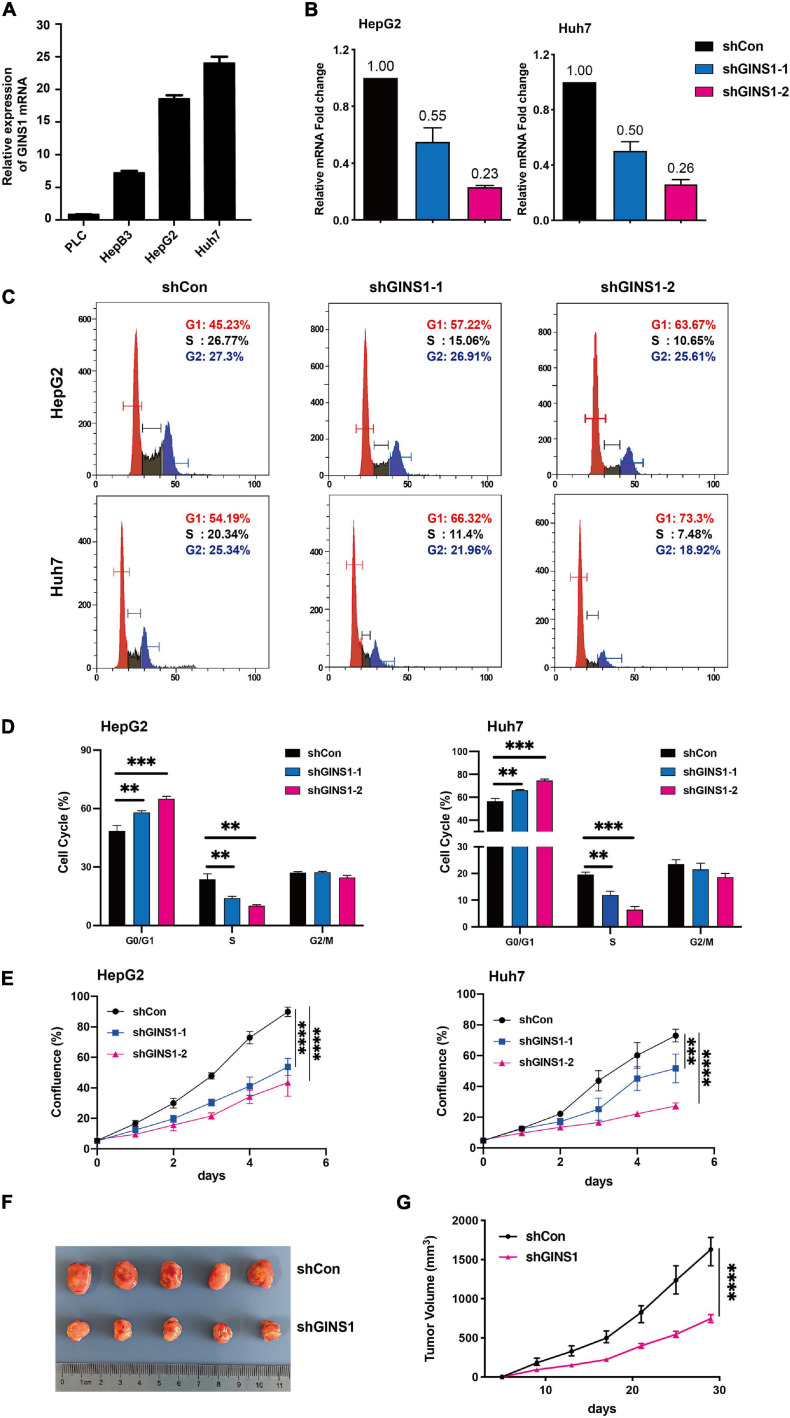
Cell cycle and proliferation regulated by GINS1 in HCC cells. **(A)** transcription level of GINS1 in HCC cells was detected by RT-qPCR. **(B)** transcription level of GINS1 in HepG2 (left panel) and Huh7 (right panel) cells stably silenced with GINS1 short hairpin RNA (shGINS1) or control shRNA (shCon) was measured by RT-qPCR. **(C,D)** cell cycles of stable transfected HepG2 and Huh7 cells were measured by flow cytometry and quantified (mean ± SD). **(E)** cell proliferations of HepG2 (upper panel) and Huh7 (lower panel) cells w/o GINS1 knockdown were assessed by IncuCyte. **(F)** representative micrograph showing tumor formation in BALB/c mice injected subcutaneously (s.c.) with Huh7 shGINS1 or shCon tumor cells. **(G)** Growth curves of the two groups in **(F)**. Data are presented as mean ± SD. ^∗∗^*P* < 0.01; ^∗∗∗^*P* < 0.001; and ^****^P < 0.0001.

The GINS1 mRNA expression was detected in HCC cell lines including PLC, HepB3, HepG2, and Huh7. HepG2 and Huh7 cells showed higher level expression of GINS1 among the four HCC cell lines (Figure 2A). The GINS1 functions in cell proliferation and cell cycle were further explored. Knockdown GINS1 by shRNAs (shGINS1-1 or shGINS1-2) evidently decreased the expression of GINS1 in HepG2 and Huh7 cells (Figure 2B). The flow cytometric analysis result of cell cycle showed that knockdown of GINS1 dramatically increased the proportion of G0/G1 phase cells and decreased the proportion of S phase cells (Figures 2C,D). Cell proliferation assay results showed that deletion of GINS1 substantially attenuated cell proliferation in HepG2 and Huh7 cells (Figure 2E). Furthermore, xenograft mouse model results showed that ablation of GINS1 inhibited tumor growth *in vivo* (Figures 2F,G). Taken together, these data suggest that silencing GINS1 induces G1/S cell cycle arrest and inhibits cell proliferation *in vitro* and *in vivo*.

### GINS1 Promotes Stem Cell Property in HCC

**FIGURE 3:**
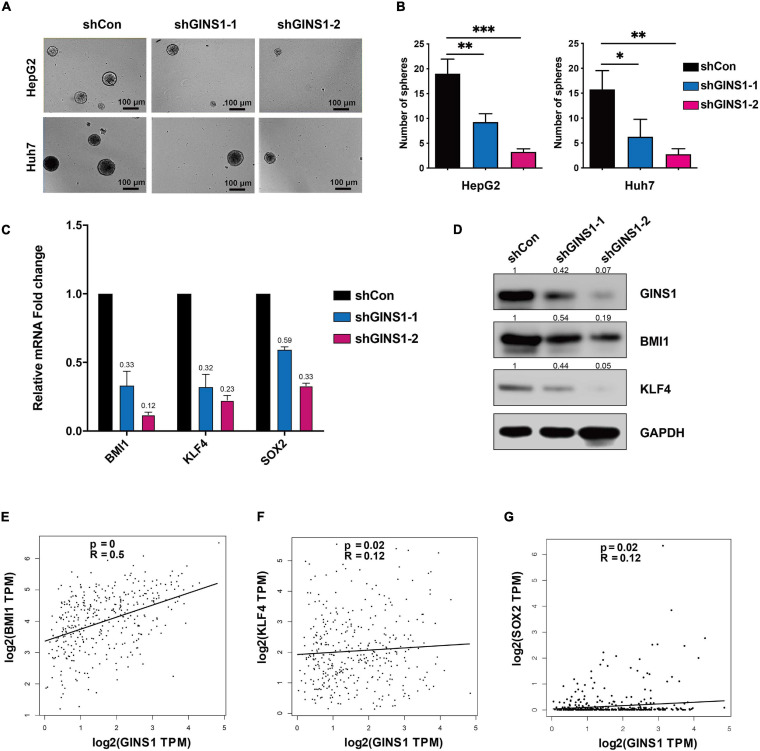
CSCs regulated by GINS1 in HCC cells. **(A,B)** images of the sphere of HepG2 and Huh7 cells w/o GINS1 ablation were captured **(A)** and the number of sphere formation was quantified **(B**; mean ± SD). Scale bar represents 100 μm. **(C)** transcription levels of CSCs markers in Huh7 shCon or shGINS1 cells were determined by RT-qPCR. **(D)** protein expression of CSCs markers in shCon or shGINS1 cells were determined by immunoblot, and the expression ratio was counted. **(E–G)** correlation of CSCs markers [BMI1 **(E)**, KLF4 **(F)**, and SOX2 **(G)**] and GINS1 expression present within TCGA database. Data are presented as mean ± SD. ^∗^*P* < 0.05; ^∗∗^*P* < 0.01; and ^∗∗∗^*P* < 0.001.

Cancer stem/initiating cells (CSCs/CICs) are well-characterized population of cells that contribute to the chemoresistance and liver cancer recurrence (Phi et al., 2018). To further investigate if GINS1 regulates liver cancer progression through CSCs, sphere formation assay was performed, and canonical cancer stem cell biomarkers were determined by qRT-PCR and western blotting, respectively. The results showed that deletion of GINS1 led to a significant reduction of sphere formation (Figures 3A,B), concurrently with down-regulation of CSCs markers including BIM1, KLF4, and SOX2 (Figures 3C,D). The Pearson analysis of TCGA database validated the positive correlation of GINS1 and CSCs biomarkers, especially BMI1 with the R superior to 0.4, in HCC tissues (Figures 3E–G). Our results suggested the stem cell property of HCC cells at least partially mediated by GINS1.

### GINS1 Induces CSCs by Activating the HRAS Pathway in HCC

**FIGURE 4:**
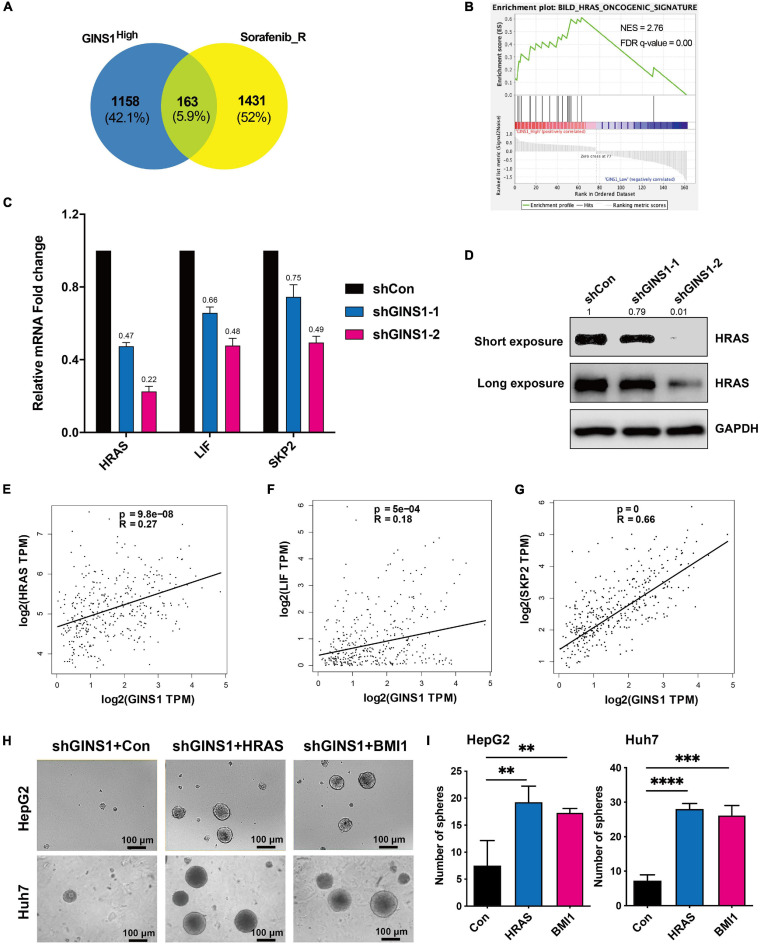
GINS1 mediated CSCs by activating the HRAS pathway. **(A)** Venn diagrams of overlapped genes in GINS1 regulation (from GSE17112) and expressed genes in response to sorafenib resistance (from GSE143233). **(B)** GSEA of the overlapped genes in **(A)** revealed HRAS pathway associated with CSCs. **(C)** transcription levels of HRAS pathway related molecules in Huh7 shGINS1 and shCon cells were determined by RT-qPCR. **(D)** protein expressions of GINS1 and HRAS in Huh7 shGINS1 and shCon cells were determined by immunoblot, and the expression ratio was counted. **(E–G)** correlation of HRAS pathway related molecules [HRAS **(E)**, LIF **(F)**, and SKP2 **(G)**] and GINS1 expression present within TCGA database. Data are presented as mean ± SD. **(H,I)** images of the sphere of HepG2 shGINS1 and Huh7 shGINS1 cells rescued with HRAS or BMI1 overexpression **(H)** and the number of sphere formation was quantified **(I**; mean ± SD). Scale bar represents 100 μm. Data are presented as mean ± SD. ^∗∗^*P* < 0.01; ^∗∗∗^*P* < 0.001; and ^****^P < 0.0001.

To identify molecular mechanisms of GINS1 in promoting HCC, functional annotation of differential expression from GSEA was conducted (Nio et al., 2017). GSE17112 which included endogenous GINS1-high-expression and GINS1-low-expression cells and GSE143233 which included 3 normal HCC patients and 3 sorafenib-resistant HCC patients were analyzed. 1321 changed genes in GINS1-high-expression group (from GSE17112) and 1594 changed genes in sorafenib-resistant group (from GSE143233) were intersected, and then 163 genes were identified (Figure 4A). Through gene enrichment analyzed by GSEA, HRAS oncogenic signature was enriched (Figure 4B). The analysis results combined GSE17112 and GSE143233 databases revealed HRAS oncogenic gene set was enriched in GINS1 high-expressed cancer cells, which displays malignant features including high proliferating activity, serial transplantation potential, and metastatic ability those are features of CSCs. We validated that members of HRAS signaling pathway such as HRAS, LIF, SKP2, were obviously down-regulated at both transcripts and proteins level in GINS1 deleted Huh7 cells (Figures 4C,D). The positive relationship of GINS1 and HRAS oncogenic pathway was also proved in HCC tissue sequencing data from TCGA, especially for BMI1 (the R superior to 0.4; Figures 4E–G). To determine whether GINS1 induced cancer stem properties through activation of HRAS pathway, the rescue assay of overexpressing HRAS pathway genes or BMI1 in HepG2 shGINS1 cells and Huh7 shGINS1 cells was performed. As shown in Figures 4H,I, rescued with HRAS or BMI1 overexpression in GINS1 depleted HCC cells led to a significant increase of sphere formation. Furthermore, overexpressing HRAS or BMI1 in GINS1 depleted cells rescued the ability of sphere formation (Figures 4H,I). These results implicated that GINS1 enhanced HCC progression through regulating HRAS signaling pathway.

### Ablation of GINS1 Reduces Sorafenib Resistance in HCC Cells

**FIGURE 5:**
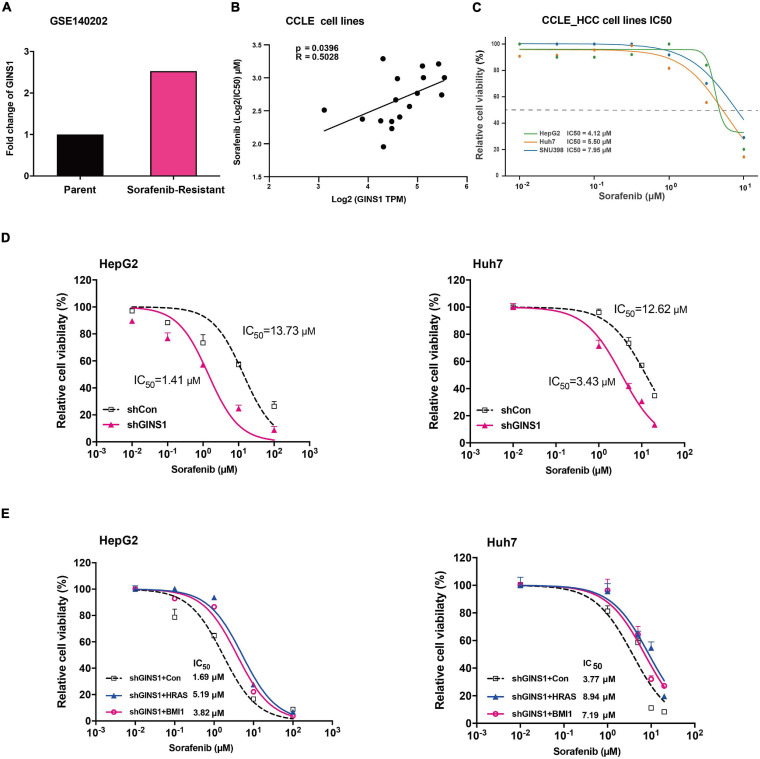
Sorafenib resistance regulated by GINS1 in HCC cells. **(A)** gene expression of GINS1 in HepG2 sorafenib-resistance and parent cell lines from GSE140202 (GEO database). **(B,C)** correlation of sorafenib IC50 and GINS1 expression in 17 hepatocellular carcinoma and colon cancer cell lines obtained from CCLE database **(B)**, and the IC50 value of HCC cell lines **(C)**. **(D)** HepG2 (left panel) and Huh7 (right panel) cells, w/o GINS1 knockdown, were treated with different doses of sorafenib as indicted and the cell viability was assessed by the MTT assay. **(E)** HepG2 shGINS1 and Huh7 shGINS1 cells, rescued with HRAS or BMI1 overexpression, treated with different doses of sorafenib as indicted and the cell viability was assessed by the MTT assay. Data are presented as mean ± SD.

Cancer stem cells are acknowledged as attribution to the chemoresistance of cancer cells (Nio et al., 2017). Sorafenib is a multi-kinase inhibitor and the only approved molecular targeted agent for advanced HCC which has shown its promising antitumor effect in a series of clinical trial (Llovet et al., 2008). However, the clinical benefit of HCC patients is limited since the rapidly development of chemoresistance. We downloaded and analyzed the RNA-seq data of sorafenib-resistance and parent cell lines from GSE140202 (GEO database), and found that GINS1 gene was up-regulated in sorafenib-resistance cells compared with parent cells in HepG2 (Figure 5A). Moreover, the IC50 values of 17 HCC and colon cancer cell lines (matched GINS1 mRNA expression and drug sensitivity data of sorafenib) were obtained from CCLE database, and showed the positive correlation between sorafenib IC50 and GINS1 expression (Figures 5B,C). To explored whether GINS1 participated in sorafenib resistance in HCC, HepG2, and Huh7 cells without or with (w/o) GINS1 deletion by shGINS1-2 were treated with increased doses of sorafenib. The cell viability results showed that knockdown of GINS1 increased sorafenib sensitivity of HCC cells in a dose-dependent manner (Figure 5D). In order to examine whether GINS1 induced sorafenib resistance is associated the stem property and HRAS pathway, HepG2 shGINS1, or Huh7 shGINS1 cells, treated with both increased doses of sorafenib and HRAS or BMI1 plasmids. The IC50 values increased by rescuing with HRAS or BMI1 overexpression in both HCC cell lines (Figure 5E). Taken together, our results suggested the sorafenib resistance of HCC cells at least partially mediated by GINS1-induced CSCs.

## Discussion

In the present study, GINS1 was identified to be highly expressed in human HCC and associated with tumor grades and predicted poor patient survival. Knocking down GINS1 arrested cell cycle and decreased tumor cells proliferation *in vitro* and *in vivo*. Moreover, GINS1 enhanced CSC property and sorafenib resistance through regulating HRAS signaling pathway.

GINS1 is a part of the tetrameric complex termed GINS, composed of SLD5, GINS2 and GINS3, and is well conserved evolutionally. It is an integral component of replicative helicase machinery and has been suggested to be involved in DNA replication. Loss of GINS1 led to embryonic lethality caused by impairing proliferation of the inner cell mass (Ueno et al., 2005). Several studies suggested that GINS played a role not only in normal cells, but also in cancer cell (Ueno et al., 2005; Bauerschmidt et al., 2007; Nakahara et al., 2010; Zhang et al., 2015). GINS complex were found to be overexpressed in breast cancer, lung cancer, etc, and high GINS1 transcriptional activity were correlated with high proliferative and metastatic activity, serial transplantation potential (Nagahama et al., 2010). In this study, silencing GINS1 inhibited cell proliferation and induced cell cycle arrest in Huh7 cells. GINS1 was also an indicator for unfavorable prognosis of HCC patients by survival analysis, indicating that GINS1 may promote the hepatocarcinogenesis and chemoresistance.

Cancer stem/initiating cells are well-characterized populations of cells that contribute to the chemoresistance and cancer recurrence. CSCs display many features of embryonic or tissue stem cells, and have the ability to self-renew, differentiate, promoting tumor growth (Reya et al., 2001). The CSC phenotype is specified and maintained by the expression of core pluripotency factors, including SRY-box 2 (SOX2), B cell-specific Moloney murine leukemia virus integration site 1 (BMI 1), Kruppel-like factor 4 (KLF4), and NANOG (Freitag et al., 2017; Shao et al., 2018; Takeda et al., 2018; Qi et al., 2019). Moreover, CSCs are inherently resistant to conventional cytotoxic therapies such as chemotherapy and radiotherapy, linking stemness to prognosis and treatment failure (Zhai and Sun, 2013). Knocking GINS1 decreased the expression of BM1, KLF4, and SOX2 in HCC cell lines, and these stem markers, especially BMI1, showed positive correlation of GINS1 in HCC tissues, indicating that the stem cell property of HCC cells at least partially mediated by GINS1.

RAS proteins are small molecular weight GTPases that couple extracellular signals to intracellular effector pathways, which play vital roles in fundamental cellular processes, including proliferation, survival, differentiation, motility, and transcription (Karnoub and Weinberg, 2008; Pylayeva-Gupta et al., 2011). In human HCC specimens, nuclear translocation of SKP2, was associated with activation of the AKT/mTOR and RAS/RAF/MAPK pathways to promote hepatocarcinogenesis *in vivo* (Delogu et al., 2015). Data of the GEO database revealed that HRAS oncogenic signature were enriched in Gins1 high-expressed cancer cells, and silencing GINS1 decreased RAS, SKP2 and LIF expression in our experiments. SKP2 is a member of the F-box family of substrate-recognition subunits of SCF ubiquitin–protein ligase complexes that has been implicated in the ubiquitin-mediated degradation of several key regulators of mammalian G1 progression (Gstaiger et al., 2001). Our study indicated that GINS1 enhanced HCC progression, cell cycle arrest, and the stem property through regulating HRAS signaling pathway.

Sorafenib is a multikinase inhibitor by inhibiting serine/threonine kinase isoforms of RAF, RAF-1, and B-RAF, leading to the inhibition of mitogen-activated protein kinase/extracellular signal-regulated kinase (ERK) signaling pathways, decreased expression of cyclin D1 and cell cycle arrest (Zhai and Sun, 2013). In our study, knocking down endogenous GINS1 with shGINS1 increased the sensitivity of HCC cells to Sorafenib, and restoring HRAS or stem associated pathway partly recovered the sorafenib resistance, suggesting the sorafenib resistance of HCC cells at least partially mediated by GINS1-induced CSCs.

In conclusion, GINS1 played a key role in cell proliferation and cell cycle in HCC. Moreover, GINS1 likely activated the RAS oncogenic pathway, promoted the stem cell activity of tumor cells, and caused sorafenib resistance. These data indicate the potential use of GINS1 in drug-resistant HCC.

## Data Availability Statement

The original contributions presented in the study are included in the article/Supplementary Material; further inquiries can be directed to the corresponding author/s.

## Ethics Statement

The animal study was reviewed and approved by Animal Experimental Ethics Committee of Peking University Cancer Hospital and Institute.

## Author Contributions

LW and SL: study design and drafting the manuscript. SL, LW, HZ, XL, ZW, and BD: biological experiments. LW, SL, HZ, and GC: bioinformatics and statistical analyses. All authors contributed to the article and approved the submitted version.

## Conflict of Interest

The authors declare that the research was conducted in the absence of any commercial or financial relationships that could be construed as a potential conflict of interest.

## Publisher’s Note

All claims expressed in this article are solely those of the authors and do not necessarily represent those of their affiliated organizations, or those of the publisher, the editors and the reviewers. Any product that may be evaluated in this article, or claim that may be made by its manufacturer, is not guaranteed or endorsed by the publisher.

## Footnotes

1
http://www.bioconductor.org

2
http://software.broadinstitute.org/gsea/index.jsp

3
http://kmplot.com/analysis/
